# Over 15 MA/cm^2^ of critical current density in 4.8 µm thick, Zr-doped (Gd,Y)Ba_2_Cu_3_O_x_ superconductor at 30 K, 3T

**DOI:** 10.1038/s41598-018-25499-1

**Published:** 2018-05-03

**Authors:** Goran Majkic, Rudra Pratap, Aixia Xu, Eduard Galstyan, Venkat Selvamanickam

**Affiliations:** Department of Mechanical Engineering, Texas Center for Superconductivity and Advanced Manufacturing Institute, University of Houston, Houston, TX 77204 USA

## Abstract

An Advanced MOCVD (A-MOCVD) reactor was used to deposit 4.8 µm thick (Gd,Y)BaCuO tapes with 15 mol% Zr addition in a single pass. A record-high critical current density (*J*_*c*_) of 15.11 MA/cm^2^ has been measured over a bridge at 30 K, 3T, corresponding to an equivalent (*I*_*c*_) value of 8705 A/12 mm width. This corresponds to a lift factor in critical current of ~11 which is the highest ever reported to the best of author’s knowledge. The measured critical current densities at 3T (B||c) and 30, 40 and 50 K, respectively, are 15.11, 9.70 and 6.26 MA/cm^2^, corresponding to equivalent *Ic* values of 8705, 5586 and 3606 A/12 mm and engineering current densities (*J*_*e*_) of 7068, 4535 and 2928 A/mm^2^. The engineering current density (*J*_*e*_) at 40 K, 3T is 7 times higher than that of the commercial HTS tapes available with 7.5 mol% Zr addition. Such record-high performance in thick films (>1 µm) is a clear demonstration that growing thick REBCO films with high critical current density (*J*_*c*_) is possible, contrary to the usual findings of strong *J*_*c*_ degradation with film thickness. This achievement was possible due to a combination of strong temperature control and uniform laminar flow achieved in the A-MOCVD system, coupled with optimization of BaZrO_3_ nanorod growth parameters.

## Introduction

Rare Earth Barium Copper Oxide (REBCO) coated conductors (CC) or 2^nd^ Generation High Temperature Superconductors (2G-HTS) have a tremendous potential for power, transport and device applications in high magnetic fields^1^. Many electric power applications like generators, motors, high energy particle accelerators, superconducting magnetic energy storage, magnetic resonance imaging and high field magnets require high engineering current densities in REBCO tapes at high magnetic fields of (2–30)T in a temperature range of (4.2–50) K^2,3^. Most of the listed applications would benefit from increase in power density, which can be achieved by increasing the engineering current density (*J*_*e*_). Additionally, a significant increase in *J*_*e*_ is perhaps the most direct way of decreasing the cost/performance ratio, which is crucial to achieving widespread utilization of 2G-HTS in applications. A significant research effort has been devoted towards improving the in-field performance, as well as self-field critical current ever since the discovery of REBCO superconductors. Introduction of nanoscale defects like BaZrO_3_(BZO)^4–8^, BaSnO_3_^9^, BaHfO_3_^10^, and Gd_3_TaO_7_^11^ in the REBCO matrix has been proven to be a powerful method to increase in-field critical current density (*J*_*c*_) through enhanced flux pinning by these defects. Studies like these have shown the pathway to realizing, until then, unthought-of levels of improving *I*_*c*_ performance in fields parallel to c-axis, or perpendicular to tape cross section.

Metal organic chemical vapor deposition (MOCVD) is one of the major deposition techniques used to produce REBCO films for 2G-HTS. Remarkable progress has been made in improving the in-field performance by thorough understanding of BZO nanorod self-assembly process and its effect on in-field performance using MOCVD. One particular aspect of interest was maximizing the lift factor (LF), defined as the ratio of *I*_*c*_ at desired temperature/field and *I*_*c*_ value at 77 K, self-field itself^12–15^. Extensive optimization of the REBCO-BZO system has been done using MOCVD, in terms of dopant concentration, film microstructure, growth conditions and composition to achieve high in-field performance^13,16–18^. However, common to most REBCO film growth techniques, *J*_*c*_ was typically found to strongly deteriorate with thickness^19–21^.

As growth of thick REBCO films with *J*_*c*_ presents a significant opportunity in improving engineering current density (*J*_*e*_) and reducing cost per ampere, the ongoing issue of strong degradation in *J*_*c*_ with thickness must be addressed. MOCVD is restricted by its inability to grow thicker films (>1 µm) in a single pass due to its poor temperature control and non-uniform flow distribution which causes a-grains to grow progressively with thickness^15,22,23^. The degradation of performance is at least in part due to this increase in fraction of a-axis oriented grains in thicker films. This results in a need for a multi-pass technique to grow thicker films^15,24^, which significantly complicates the process.

In a previous study, an Advanced Metal Organic Chemical Vapor Deposition (A-MOCVD) system was developed under the ARPA-E Grid-Scale Rampable Intermittent Dispatchable Storage (GRIDS) program, with a goal of addressing the critical issues found with conventional MOCVD reactors^22^. The A-MOCVD uses direct ohmic heating of a suspended substrate tape and direct temperature monitoring using non-contact optical probes^22^. A highly laminar and uniform flow has been achieved in the direction perpendicular to tape using a new vapor path design^22,23^. Previously, near 1000 A/12 mm critical current was achieved in 1.8 µm thick undoped REBCO on IBAD-MgO/LMO substrate in a single pass deposition using the A-MOCVD system^22^. Furthermore, a record high lift factor of 9 was achieved in a 2 µm thick 20 mol% Zr doped REBCO film, at 30 K, 2.5 T (B||c), deposited in a single pass^23^. In addition, up to 4 µm thick undoped REBCO films have been grown without any a-grains in a single pass deposition using the A-MOCVD system^22^. The flow uniformity, coupled with low thermal mass of the heated tape compared to use of susceptor, as well as closed loop temperature control using optical emission probes enabled growth of thick films without texture degradation.

In this study, we use the A-MOCVD reactor to grow 4.8 µm thick 15 mol% Zr-doped REBCO films in a single pass over a 30 cm long flexible hastelloy/IBAD MgO/LMO substrate. The deposition rate in A-MOCVD was 0.192 µm/min compared with conventional MOCVD deposition rate of 0.1 µm/min due to higher process efficiency of A-MOCVD^22^. The main objective of this study is to investigate if the capability to grow thick films can be coupled with optimization of BZO nanorod growth using the A-MOCVD system to provide very thick, high quality films optimized for in-field performance. If this goal is achieved, it will greatly simplify the deposition process of thick films due to single pass deposition compared to conventional MOCVD. In addition, if the same high performance is maintained throughout the thickness as in lower thickness tapes^13,15,23^, it will enhance the engineering current density (*J*_*e*_) significantly, which will in turn decrease the cost/performance ratio.

## Results and Discussion

**Figure 1:**
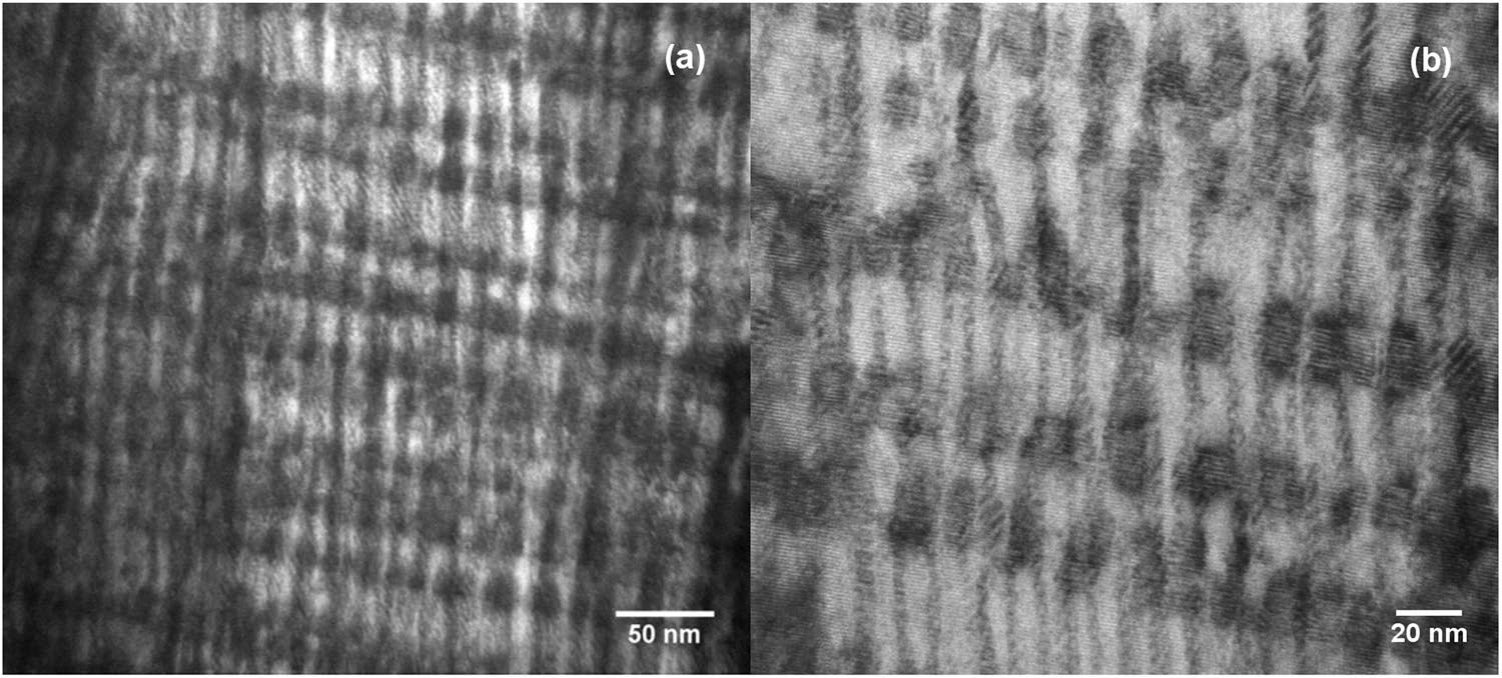
Cross-section TEM microstructure of a 4.8 µm thick REBCO tape with 15 mol% Zr addition at two magnifications, showing well-aligned BaZrO_3_ nano columns interspersed with RE_2_O_3_ precipitates.

Figure 1 shows cross section microstructure of the 4.8 μm thick film of (Gd,Y)BCO with 15 mol% Zr addition made in a single pass in A-MOCVD. An excellent alignment of the BZO nanorods was observed over the entire film thickness. This microstructure is in contrast to that of a 3.2 µm thick 20% Zr-added GdYBCO film made in three passes by conventional MOCVD by our group^24^. In that case, the length of the BZO nanorod was found to be reduced with increasing REBCO layer thickness and a low density of thick and short BZO nanorods was observed in the 100–200 nm interface between two passes. These features were reported to be potential reasons for the lower *J*_*c*_ of the 3.2 µm thick Zr-added GdYBCO film compared to a 0.9 µm thick Zr-added GdYBCO film. Presence of RE_2_O_3_ precipitates along ab plane, as shown in Fig. 1, was also observed over the entire cross-section. It should be noted that due to growth optimization as mentioned in^23^, these precipitates do not affect BZO nanorod growth along c-axis, which was reported as an issue in non-optimized films^25^.

**Figure 2:**
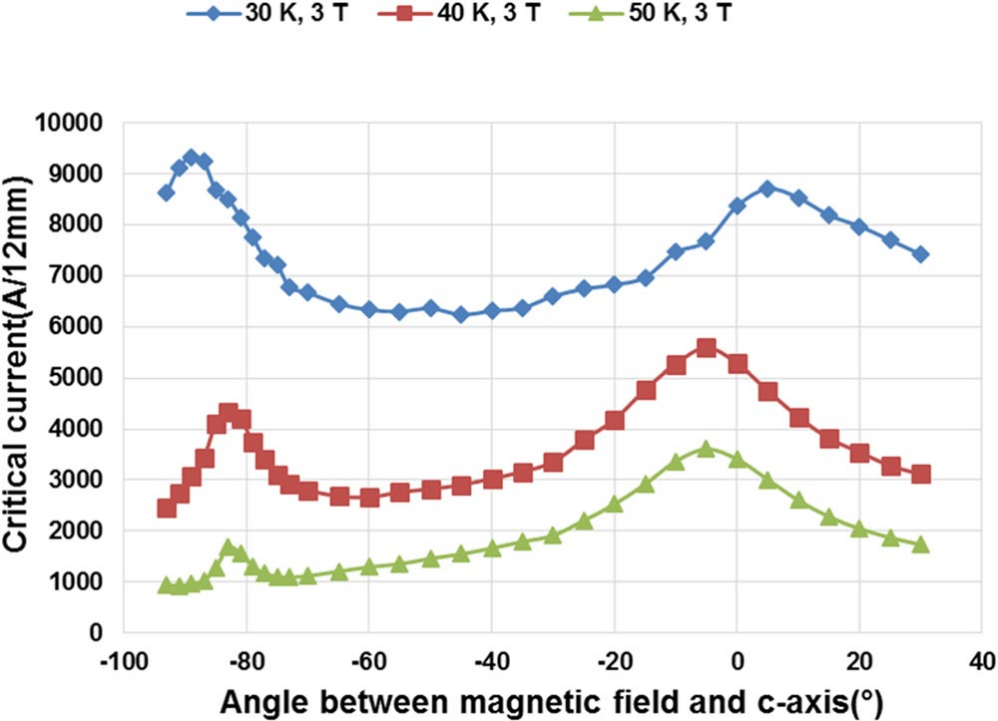
Angular dependence of critical current of a 4.8 µm REBCO tape with 15 mol% Zr addition at 30 K, 40 K and 50 K in 3T magnetic field.

The *I*_*c*_ angular dependence at 30 K, 40 K and 50 K in 3T magnetic field is shown in Fig. 2. A record high critical current value of 8705 A/12 mm has been achieved at 30 K, 3T(B||c) which is more than two times higher than the best value of 3963 A/12 mm obtained in 2.2 µm thick, 20 mol% Zr added GdYBCO film processed in two passes using conventional MOCVD^15^. This value is also 2.7 times higher than the previously achieved critical current value obtained using A-MOCVD in a 2 µm thick, 20 mol% Zr added GdYBCO film processed in a single pass. Also, it is more than 7 times higher than the critical current value of 1072 A/12 mm obtained in 0.9 µm thick, 15 mol% Zr added GdYBCO film made by conventional MOCVD^26^. The corresponding lift factor in critical current at 30 K, 3T is 10.89 which is a record high value ever reported to the best of author’s knowledge.

The critical current density at 30 K, 3T is 15.11 MA cm^−2^, which is about the same as the highest value of 15 MA cm^−2^ attained in 2.2 µm thick, 20 mol% Zr added GdYBCO film processed in two passes^15^. This *J*_c_ value also compares very well with the record high *J*_c_ value of 20.1 MA cm^−2^ reported at 30 K, 3T in 0.9 µm thick, 25 mol% Zr added GdYBCO film made by conventional MOCVD^13,15^. The *J*_*c*_ results are summarized in Table 1.

**Table 1:** Comparison of performance and deposition details between this work done using A-MOCVD and previous results obtained using conventional MOCVD.

Ref.	System	No. of passes	REBCO thickness [µm]	Mol% of Zr in precursor	IC/12 mm, 30 K, 3T [A]	JC, 30 K, 3T [MA/cm^2^]
^13^	MOCVD	1	0.9	25	2195	20.32
^15^	MOCVD	2	2.2	20	3963	15.01
This work	A-MOCVD	1	4.8	15	8705	15.11

**Figure 3:**
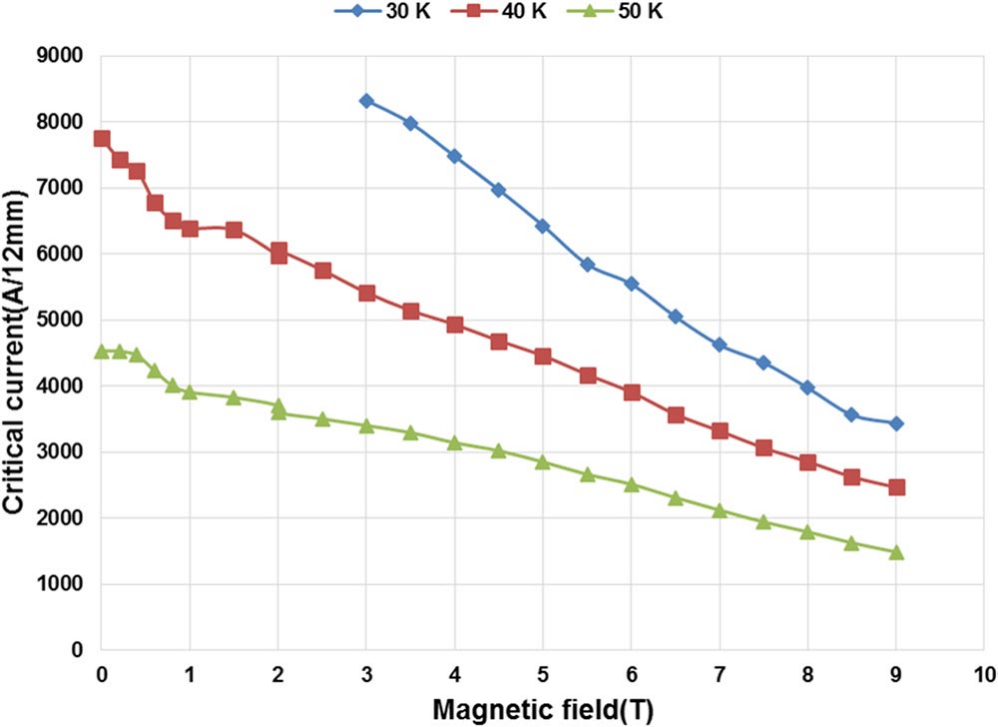
Critical current of a 4.8 µm REBCO tape with 15 mol% Zr addition in magnetic fields up to 9T applied along the c-axis at 30 K, 40 K and 50 K.

Shown in Fig. 3 is the critical current in magnetic fields up to 9T applied along the c-axis at 30, 40 and 50 K. At 3T, record high critical current values of 5586 A/12 mm and 3606 A/12 mm have been measured at 40 K and 50 K, respectively, which is 1.9 times higher than the best reported value in 2.2 µm thick, 20 mol% Zr added GdYBCO film processed in two passes using conventional MOCVD^15^. The corresponding *J*_c_ values are 9.69 MA cm^−2^ and 6.26 MA cm^−2^ at 40 K and 50 K respectively.

The engineering current density (*J*_*e*_) values (considering a typical 40 µm thick copper stabilizer) at 30 K, 40 K and 50 K in magnetic field 3T(B||c) are 7068 A/mm^2^, 4535 A/mm^2^ and 2928 A/mm^2^ respectively. The engineering current density at 40 K, 3T is a factor of 1.9 times higher than the best value of 2360 A/mm^2^ reported in the 2.2 µm thick, 20 mol% Zr added GdYBCO film and more than 7 times higher than the best commercial HTS tapes reported in a recent study^15^. Such high *J*_*e*_ values offer an opportunity for a significant reduction in the amount of superconductor tape needed for superconducting devices which in turn reduces the cost for commercial applications.

The minimum value of critical current (*I*_c,min_) in angular dependence measurements is an important parameter since a wide range of field orientations can be present in superconducting coils near the edges. The *I*_c,min_ value at 30 K, 3T is 6238 A/12 mm, which is 1.9 times higher than *I*_c,min_ value of 2.2 µm thick 20 mol% Zr added GdYBCO film grown by conventional MOCVD^15^. Therefore, the entire angular Ic dependence shown in Fig. 2 is about a factor of two higher than that reported in^15^.

**Figure 4:**
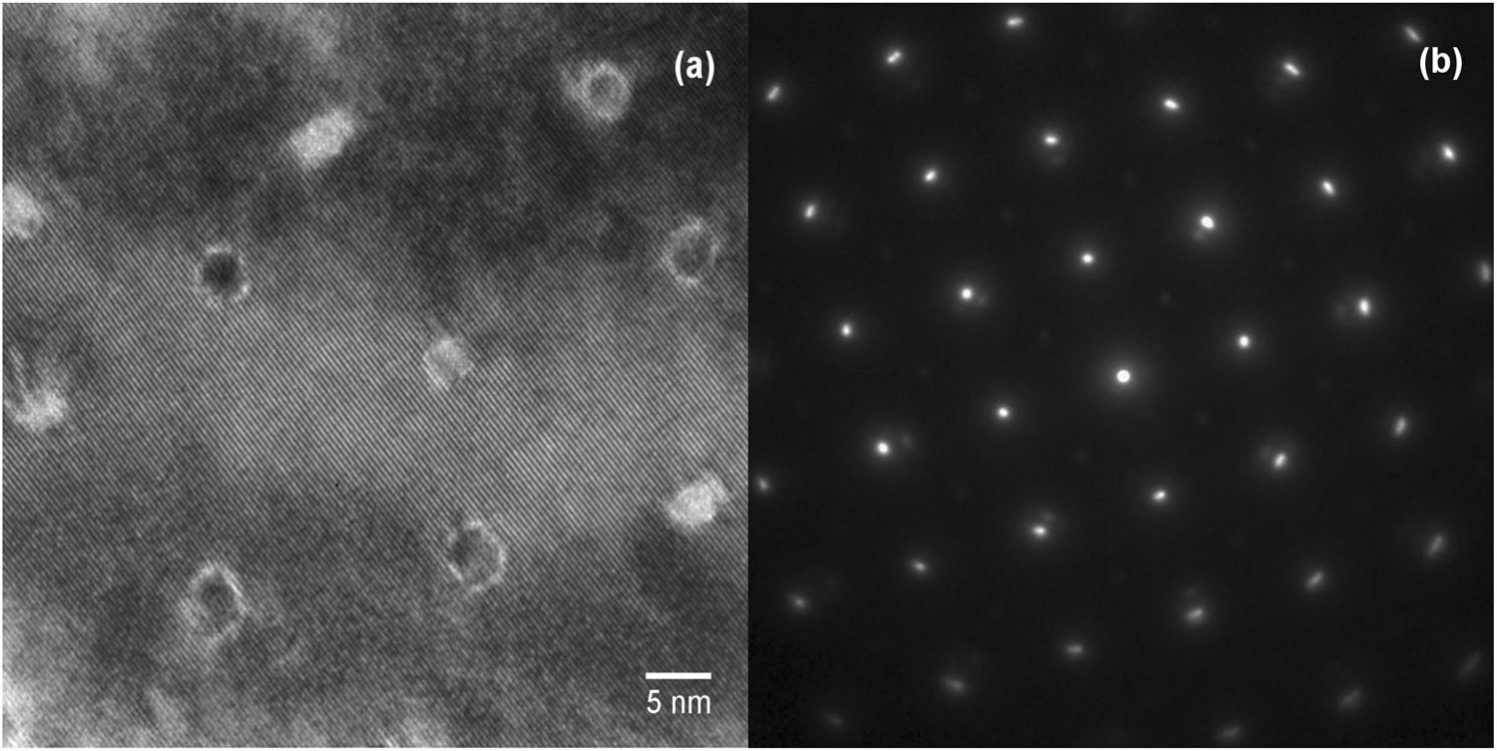
TEM plan-view analysis: (**a**) microstructure and (**b**) plan-view electron diffraction pattern, revealing BZO nanorods with ~3.5 and 17.5 nm diameter and spacing, respectively, and sharp in-plane texture.

Figure 4 shows plane-view TEM micrograph and the corresponding electron diffraction pattern (ab plane) of the 4.8 µm thick, 15 mol% Zr-added GdYBCO film. The average diameter and spacing of the BZO nanorods was found to be ~3.5 and 17.5 nm, respectively, which is consistent with that observed before in 2 μm thick films processed in A-MOCVD^23^. It has been established that long continuous and small diameter BZO nanorods that permeate the whole film contribute to strong pinning and high lift factor in critical current^12–14,16,23^. Such dependence of in-field behavior on nanorod morphology has also been reported in^27^. The in-plane texture of the film appears very sharp as can be seen in the diffraction pattern, indicating a good balance between high lift factor and *I*_*c*_ at 77 K, 0T. The deterioration of in-plane texture in REBCO films with BZO nanorods of very small diameter has been reported as a factor that ultimately leads to degradation in performance^23^. Our results show that using A-MOCVD fine BZO nanorods can be obtained for strong pinning without sacrificing texture quality.

**Figure 5:**
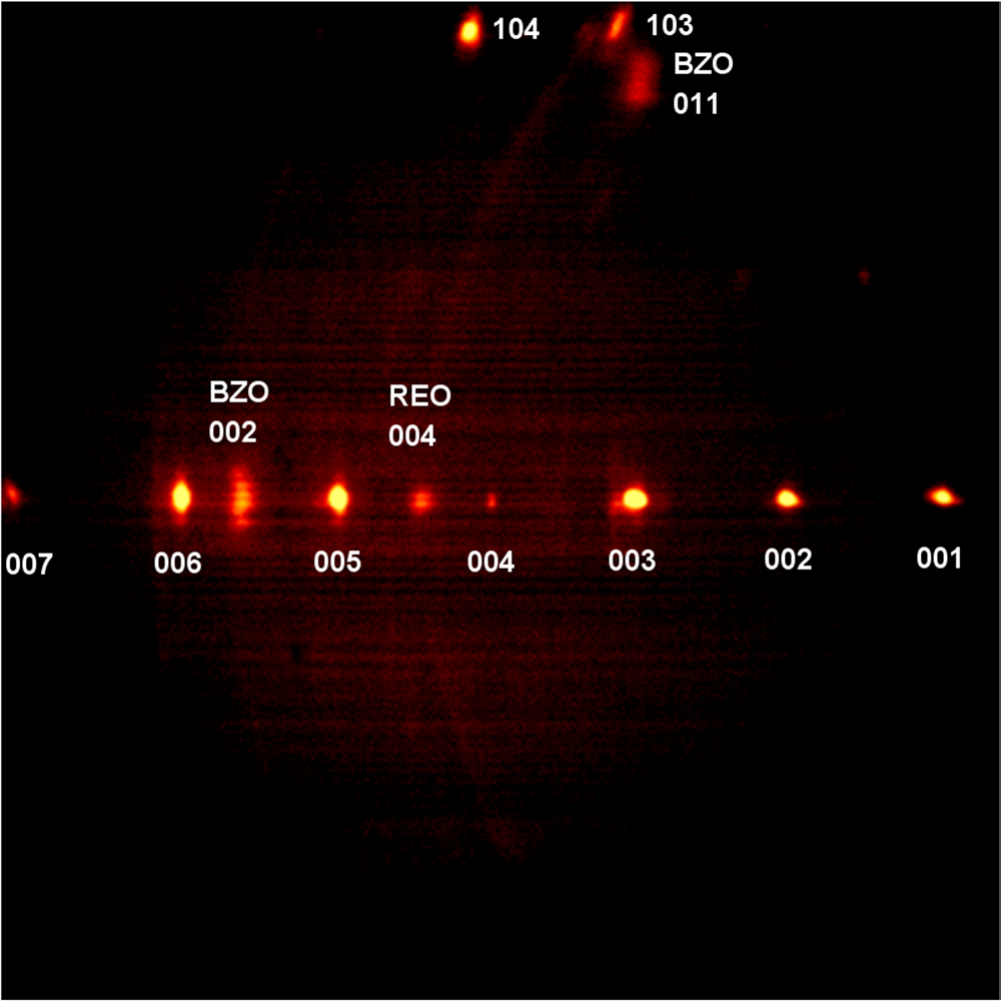
2D-XRD diffraction pattern of 4.8 µm thick REBCO tape with 15 mol% Zr addition processed by A-MOCVD in a single pass. BZO (011) peak is streaking in the direction perpendicular to nanorod length indicating a small nanorod diameter.

Figure 5 shows a two dimensional diffraction pattern of as-processed, non-annealed REBCO film, obtained using Bruker GADDS system. The pattern reveals highly-textured c-axis oriented REBCO peaks (001 and 101 series), BZO (002 and 101) and RE_2_O_3_ 004. Re_2_O_3_ has been deliberately introduced through excess RE to form ab-plane precipitates^8^, but with optimization to prevent plate-like RE_2_O_3_ formation that interrupts BZO nanorod growth along c axis^25^. Film thickness can also be estimated from the intensity of hastelloy substrate rings, as was discussed in^23^, which are almost invisible in this case, compared to that reported in refs^22,23,26^, indicating a very thick REBCO film. It has also been established that the streaking of BZO 101 peak in the direction perpendicular to REBCO 001 indicates a small diameter of nanorods, which is similar to the observation in^23^. The diffraction pattern in Fig. 5 clearly indicates very good texture for a 4.8 µm, highly Zr-doped film, processed in a single pass. There is no evidence of significant broadening of any of the REBCO 001 peaks, which indicates a very good out-of-plane texture.

## Summary

An advanced MOCVD (A-MOCVD) reactor has been used to deposit 4.8 µm thick, 15 mol% Zr doped (Gd,Y)BaCuO tape in a single pass. It is remarkable that a nearly 5 µm thick film has been grown in a single pass without any degradation in texture or BZO nanorod alignment, which simplifies the deposition and reduces the processing cost significantly. Record-high critical currents of 8705 A/12 mm, 5586 A/12 mm and 3606 A/12 mm have been obtained at 30 K, 40 K and 50 K respectively in a magnetic field of 3T (B||c) which are approximately two times higher than the best value reported in literature. The corresponding engineering current density (*J*_*e*_) values are 7068 A/mm^2^, 4535 A/mm^2^ and 2928 A/mm^2^ at 30 K, 40 K and 50 K respectively. The engineering current density (*J*_*e*_) of this tape at 40 K, 3T is more than 7 times higher than the best commercial HTS tapes reported in recent study. Such a high performance opens the way to meet the need for low-cost HTS tapes (in terms of $/kA-m) for HTS applications.

## Methods

### Sample preparation

A-MOCVD was used for depositing 4.8 µm thick REBCO film in a single pass on a 12 mm wide, 50 µm Hastelloy C-276 substrate with biaxially textured IBAD-MgO templates and LaMnO_3_ cap layer^28,29^. After REBCO film deposition, ~3 µm thick silver layer was deposited on top of it as a protection and current contact layer.

### Measurements

Transport *I*_*c*_ measurements were performed using a four-probe method and *I*_*c*_ was defined at a voltage criterion of 1 μV/cm. In-field *I*_*c*_ measurements were performed in a 9T solenoid system over temperature and field ranges of 30 K to 50 K and 0 to 9T, respectively, and over the angular range of −105° to 35° relative to the tape normal direction. A bridge of 0.2–0.3 mm in width and approximately 10 mm in length was used for critical current measurements to minimize the required current and sample heating. Focused ion beam milling was used to prepare cross section to measure the thickness of the film. Transmission Electron Microscopy (TEM) was performed using JEOL 2000FX to examine plan and cross section views of the sample to study morphology, orientation and size of nanoscale defects created in the film. Diffraction measurements were performed on a Bruker AXS General Area Detector Diffraction System (GADDS).

### Data Availability Statement

All data generated or analyzed during this study are included in this published article.
